# On the Versatility of Open Logical Relations: Continuity, Automatic Differentiation, and a Containment Theorem

**DOI:** 10.1007/978-3-030-44914-8_3

**Published:** 2020-04-18

**Authors:** Gilles Barthe, Raphaëlle Crubillé, Ugo Dal Lago, Francesco Gavazzo

**Affiliations:** ETH Zurich, Zurich, Switzerland; 9 MPI for Security and Privacy, Bochum, Germany; 10 University of Bologna, Bologna, Italy; 11 INRIA Sophia Antipolis, Sophia Antipolis, France; 12 IMDEA Software Institute, Madrid, Spain

**Keywords:** Lambda Calculus, Logical Relations, Continuity Analysis, Automatic Differentiation

## Abstract

Logical relations are one among the most powerful techniques in the theory of programming languages, and have been used extensively for proving properties of a variety of higher-order calculi. However, there are properties that cannot be immediately proved by means of logical relations, for instance program continuity and differentiability in higher-order languages extended with real-valued functions. Informally, the problem stems from the fact that these properties are naturally expressed on terms of non-ground type (or, equivalently, on open terms of base type), and there is no apparent good definition for a base case (i.e. for closed terms of ground types). To overcome this issue, we study a generalization of the concept of a logical relation, called *open logical relation*, and prove that it can be fruitfully applied in several contexts in which the property of interest is about expressions of first-order type. Our setting is a simply-typed $\lambda$-calculus enriched with real numbers and real-valued first-order functions from a given set, such as the one of continuous or differentiable functions. We first prove a containment theorem stating that for any collection of real-valued first-order functions including projection functions and closed under function composition, any well-typed term of first-order type denotes a function belonging to that collection. Then, we show by way of open logical relations the correctness of the core of a recently published algorithm for forward automatic differentiation. Finally, we define a refinement-based type system for local continuity in an extension of our calculus with conditionals, and prove the soundness of the type system using open logical relations.

* The Second and Fourth Authors are supported by the ANR project 16CE250011 REPAS, the ERC Consolidator Grant DIAPASoN – DLV-818616, and the MIUR PRIN 201784YSZ5 ASPRA.

## 1 Introduction

Logical relations have been extremely successful as a way of proving equivalence between concrete programs as well as correctness of program transformations. In their "unary" version, they also are a formidable tool to prove termination of typable programs, through the so-called *reducibility* technique. The class of programming languages in which these techniques have been instantiated includes not only higher-order calculi with simple types, but also calculi with recursion [3,2,23], various kinds of effects [14,12,25,36,10,11,34], and concurrency [56,13].

Without any aim to be precise, let us see how reducibility works, in the setting of a simply typed calculus. The main idea is to define, by induction on the structure of types, the concept of a well-behaved program, where in the base case one simply makes reference to the underlying notion of observation (e.g. being strong normalizing), while the more interesting case is handled by stipulating that reducible higher-order terms are those which maps reducible terms to reducible terms, this way exploiting the inductive nature of simple types. One can even go beyond the basic setting of simple types, and extend reducibility to, e.g., languages with recursive types [23,2] or even untyped languages [44] by means of techniques such as step-indexing [3].

The same kind of recipe works in a relational setting, where one wants to *compare* programs rather than merely *proving properties* about them. Again, two terms are equivalent at base types if they have the same observable behaviour, while at higher types one wants that equivalent terms are those which maps equivalent arguments to equivalent results.

There are cases, however, in which the property one observes, or the property in which the underlying notion of program equivalence or correctness is based, is formulated for types which are *not* ground (or equivalently, it is formulated for open expressions). As an example, one could be interested in proving that in a higher-order type system all *first-order* expressions compute numerical functions of a specific kind, for example, continuous or derivable ones. We call such properties *first-order properties*[5]. As we will describe in Section 3 below, logical relations do not seem to be applicable *off-the-shelf* to these cases. Informally, this is due to the fact that we cannot start by defining a base case for ground types and then build the relation inductively.

In this paper, we show that logical relations and reducibility can deal with first-order properties in a compositional way without altering their nature. The main idea behind the resulting definition, known as *open logical relations* [59], consists in parameterizing the set of related terms of a certain type (or the underlying reducibility set) on a *ground environment*, this way turning it into a set of pairs of *open terms*. As a consequence, one can define the target first-order property in a natural way.

[5] To avoid misunderstandings, we emphasize that we use first-order properties to refer to properties of expressions of first-order types—and not in relation with definability of properties in first-order predicate logic.

Generalizations of logical relations to open terms have been used by several authors, and in several (oftentimes unrelated) contexts (see, for instance, [15,39,47,30,53]). In this paper, we show how open logical relations constitute a powerful technique to systematically prove first-order properties of programs. In this respect, the paper's technical contributions are applications of open logical relations to three distinct problems.

- In Section 4, we use open logical relations to prove a general Containment Theorem. Such a theorem serves as a vehicle to introduce open logical relations but is also of independent interest. The theorem states that given a collection $\mathfrak{F}$ of real-valued functions including projections and closed under function composition, any first-order term of a simply-typed $\lambda$-calculus endowed with primitives for real numbers and operators computing functions in $\mathfrak{F}$, computes itself a function in $\mathfrak{F}$. As an instance of such a result, we see that any first-order term in a simply-typed $\lambda$-calculus extended with primitives for continuous functions, computes a continuous function. Although the Containment Theorem can be derived from previous results by Lafont [41] (see Section 7), our proof is purely syntactical and consists of a straightforward application of open logical relations.
- In Section 5, we use open logical relations to prove correctness of a core algorithm for forward automatic differentiation of simply-typed terms. The algorithm is a fragment of the one presented in [50]. More specifically, any first-order term is proved to be mapped to another first-order term computing its derivative, in the usual sense of mathematical analysis. This goes beyond the Containment Theorem by dealing with relational properties.
- In Section 6, we consider an extended language with an if-then-else construction. When dealing with continuity, the introduction of conditionals invalidates the Containment Theorem, since conditionals naturally introduce discontinuities. To overcome this deficiency, we introduce a refinement type system ensuring that first-order typable terms are continuous functions on some intended domain, and use open logical relations to prove the soundness of the type system.

Due to space constraints, many details have to be omitted, but can be found in an Extended Version of this work [7].

## 2 The Playground

In order to facilitate the communication of the main ideas behind open logical relations and their applications, this paper deals with several vehicle calculi. All such calculi can be seen as derived from a unique calculus, denoted by $\Lambda^{\times,\to,\mathtt{R}}$, which thus provides the common ground for our inquiry. The calculus $\Lambda^{\times,\to,\mathtt{R}}$ is obtained by adding to the simply typed $\lambda$-calculus with product and arrow types (which we denote by $\Lambda^{\times,\to}$) a ground type $\mathtt{R}$ for real numbers and constants $\underline{r}$ of type $\mathtt{R}$, for each real number $r$.

Given a collection $\mathfrak{F}$ of real-valued functions, i.e. functions $f : \mathbb{R}^n \to \mathbb{R}$ (with $n \geq 1$), we endow $\Lambda^{\times,\to,\mathtt{R}}$ with an operator $\underline{f}$, for any $f \in \mathfrak{F}$, whose intended meaning is that whenever $t_1, \ldots, t_n$ compute real numbers $r_1, \ldots, r_n$, then $\underline{f}(t_1, \ldots, t_n)$ compute $f(r_1, \ldots, r_n)$. We call the resulting calculus $\Lambda_{\mathfrak{F}}^{\times,\to,\mathtt{R}}$. Depending on the application we are interested in, we will take as $\mathfrak{F}$ specific collections of real-valued functions, such as continuous or differentiable functions.

The syntax and static semantics of $\Lambda_{\mathfrak{F}}^{\times,\to,\mathtt{R}}$ are defined in Figure 1, where $f : \mathbb{R}^n \to \mathbb{R}$ belongs to $\mathfrak{F}$. The static semantics of $\Lambda_{\mathfrak{F}}^{\times,\to,\mathtt{R}}$ is based on judgments of the form $\Gamma \vdash t : \tau$, which have the usual intended meaning. We adopt standard syntactic conventions as in [6], notably the so-called variable convention. In particular, we denote by $FV(t)$ the collection of free variables of $t$ and by $s[t/x]$ the capture-avoiding substitution of the expression $t$ for all free occurrences of $x$ in $s$.

$$\tau ::= \mathtt{R} \mid \tau \times \tau \mid \tau \to \tau \qquad\qquad \Gamma ::= \cdot \mid x : \tau, \Gamma$$

$$t ::= x \mid \underline{r} \mid \underline{f}(t, \ldots, t) \mid \lambda x.t \mid tt \mid (t, t) \mid t.1 \mid t.2$$

$$\frac{}{\Gamma, x : \tau \vdash x : \tau} \qquad \frac{}{\Gamma \vdash \underline{r} : \mathtt{R}} \qquad \frac{\Gamma \vdash t_1 : \mathtt{R} \quad \cdots \quad \Gamma \vdash t_n : \mathtt{R}}{\Gamma \vdash \underline{f}(t_1, \ldots, t_n) : \mathtt{R}} \qquad \frac{\Gamma, x : \tau_1 \vdash t : \tau_2}{\Gamma \vdash \lambda x.t : \tau_1 \to \tau_2}$$

$$\frac{\Gamma \vdash s : \tau_1 \to \tau_2 \quad \Gamma \vdash t : \tau_1}{\Gamma \vdash st : \tau_2} \qquad \frac{\Gamma \vdash t_1 : \tau \quad \Gamma \vdash t_2 : \sigma}{\Gamma \vdash (t_1, t_2) : \tau \times \sigma} \qquad \frac{\Gamma \vdash t : \tau_1 \times \tau_2}{\Gamma \vdash t.i : \tau_i}\ (i \in \{1, 2\})$$

Fig. 1: Static semantics of $\Lambda_{\mathfrak{F}}^{\times,\to,\mathtt{R}}$.

We do not confine ourselves with a fixed operational semantics (e.g. with a call-by-value operational semantics), but take advantage of the simply-typed nature of $\Lambda_{\mathfrak{F}}^{\times,\to,\mathtt{R}}$ and opt for a set-theoretic denotational semantics. The category of sets and functions being cartesian closed, the denotational semantics of $\Lambda_{\mathfrak{F}}^{\times,\to,\mathtt{R}}$ is standard and associates to any judgment $x_1 : \tau_1, \ldots, x_n : \tau_n \vdash t : \tau$, a function $[\![x_1 : \tau_1, \ldots, x_n : \tau_n \vdash t : \tau]\!] : \prod_i [\![\tau_i]\!] \to [\![\tau]\!]$, where $[\![\tau]\!]$—the *semantics* of $\tau$—is thus defined:

$$[\![\mathtt{R}]\!] = \mathbb{R}; \qquad [\![\tau_1 \to \tau_2]\!] = [\![\tau_2]\!]^{[\![\tau_1]\!]}; \qquad [\![\tau_1 \times \tau_2]\!] = [\![\tau_1]\!] \times [\![\tau_2]\!].$$

Due to space constraints, we omit the definition of $[\![\Gamma \vdash t : \tau]\!]$ and refer the reader to any textbook on the subject (such as [43]).

## 3 A Fundamental Gap

In this section, we will look informally at a problem which, apparently, cannot be solved using vanilla reducibility or logical relations. This serves both as a motivating example and as a justification of some of the design choices we had to do when designing open logical relations.

Consider the simply-typed $\lambda$-calculus $\Lambda^{\times,\to}$, the prototypical example of a well-behaved higher-order functional programming language. As is well known, $\Lambda^{\times,\to}$ is strongly normalizing and the technique of logical relations can be applied on-the-nose. The proof of strong normalization for $\Lambda^{\times,\to}$ is structured around the definition of a family of reducibility sets of *closed* terms $\{Red_\tau\}_\tau$, indexed by types. At any atomic type $\tau$, $Red_\tau$ is defined as the set of terms (of type $\tau$) having the property of interest, i.e. as the collection of strongly normalizing terms. The set $Red_{\tau_1\to\tau_2}$, instead, contains those terms which, when applied to a term in $Red_{\tau_1}$, returns a term in $Red_{\tau_2}$. Reducibility sets are *afterwards* generalised to open terms, and finally all typable terms are shown to be reducible.

Let us now consider the calculus $\Lambda_{\mathfrak{F}}^{\times,\to,\mathtt{R}}$, where $\mathfrak{F}$ contains the addition and multiplication functions only. This language has already been considered in the literature, under the name of *higher-order polynomials* [22,40], which are crucial tools in higher-order complexity theory and resource analysis. Now, let us ask ourselves the following question: can we say anything about the nature of those functions $\mathbb{R}^n \to \mathbb{R}$ which are denoted by (closed) terms of type $\mathtt{R}^n \to \mathtt{R}$? Of course, all the polynomials on the real field can be represented, but can we go beyond, thanks to higher-order constructions? The answer is negative: terms of type $\mathtt{R}^n \to \mathtt{R}$ represent all *and only* the polynomials [5,17]. This result is an instance of the general containment theorem mentioned at the end of Section 1.

Let us now focus on proofs of this containment result. It turns out that proofs from the literature are not compositional, and rely on"heavyweight" tools, including strong normalization of $\Lambda^{\times,\to}$ and soundness of the underlying operational semantics. In fact, proving the result using usual reducibility arguments would not be immediate, precisely because there is no obvious choice for the base case. If, for example, we define $Red_{\mathtt{R}}$ as the set of terms strongly normalizing to a numeral, $Red_{\mathtt{R}^n\to\mathtt{R}}$ as the set of polynomials, and for any other type as usual, we soon get into troubles: indeed, we would like the two sets of functions

$$Red_{\mathtt{R}\times\mathtt{R}\to\mathtt{R}}; \qquad\qquad Red_{\mathtt{R}\to(\mathtt{R}\to\mathtt{R})};$$

to denote *essentially* the same set of functions, modulo the adjoint between $\mathbb{R}^2 \to \mathbb{R}$ and $\mathbb{R} \to (\mathbb{R} \to \mathbb{R})$. But this is clearly not the case: just consider the function $f$ in $\mathbb{R} \to (\mathbb{R} \to \mathbb{R})$ thus defined:

$$f(x) = \begin{cases} \lambda y.y & \text{if } x \geq 0 \\ \lambda y.y + 1 & \text{if } x < 0. \end{cases}$$

Clearly, $f$ turns any *fixed* real number to a polynomial, but when curried, it is far from being a polynomial. In other words, reducibility seems apparently inadequate to capture situations like the one above, in which the "base case" is not the one of *ground* types, but rather the one of *first-order* types.

Before proceeding any further, it is useful to fix the boundaries of our investigation. We are interested in proving that (the semantics of) programs of first-order type $\mathtt{R}^n \to \mathtt{R}$ enjoy first-order properties, such as continuity or differentiability, under their standard interpretation in calculus and real analysis. More specifically, our results do not cover notions of continuity and differentiability studied in fields such as (exact) real-number computation [57] or computable analysis [58], which have a strong domain-theoretical flavor, and higher-order generalizations of continuity and differentiability (see, e.g., [26,27,32,29]). We leave for future work the study of open logical relations in these settings. What this paper aims to provide, is a family of *lightweight* techniques that can be used to show that practical properties of interest of real-valued functions are guaranteed to hold when programs are written taking advantage of higher-order constructors. We believe that the three case studies we present in this paper are both a way to point to the practical scenarios we have in mind and of witnessing the versatility of our methodology.

## 4 Warming Up: A Containment Theorem

In this section we introduce open logical relations in their unary version (i.e. open logical predicates). We do so by proving the following Containment Theorem.

**Theorem 1 (Containment Theorem).** *Let $\mathfrak{F}$ be a collection of real-valued functions including projections and closed under function composition. Then, any $\Lambda_{\mathfrak{F}}^{\times,\to,\mathtt{R}}$ term $x_1 : \mathtt{R}, \ldots, x_n : \mathtt{R} \vdash t : \mathtt{R}$ denotes a function (from $\mathbb{R}^n$ to $\mathbb{R}$) in $\mathfrak{F}$. That is, $[\![x_1 : \mathtt{R}, \ldots, x_n : \mathtt{R} \vdash t : \mathtt{R}]\!] \in \mathfrak{F}$.*

As already remarked in previous sections, notable instances of Theorem 1 are obtained by taking $\mathfrak{F}$ as the collection of continuous functions, or as the collection of polynomials.

Our strategy to prove Theorem 1 consists in defining a logical predicate, denoted by $\mathcal{F}$, ensuring the denotation of programs of a first-order type to be in $\mathfrak{F}$, and hereditary preserving this property at higher-order types. However, $\mathfrak{F}$ being a property of real-valued functions—and the denotation of an *open* term of the form $x_1 : \mathtt{R}, \ldots, x_n : \mathtt{R} \vdash t : \mathtt{R}$ being such a function—we shall work with open terms with free variables of type $\mathtt{R}$ and parametrize the candidate logical predicate by types *and* environments $\Theta$ containing such variables.

This way, we obtain a family of logical predicates $\mathcal{F}_\tau^\Theta$ acting on terms of the form $\Theta \vdash t : \tau$. As a consequence, when considering the ground type $\mathtt{R}$ and an environment $\Theta = x_1 : \mathtt{R}, \ldots, x_n : \mathtt{R}$, we obtain a predicate $\mathcal{F}_\mathtt{R}^\Theta$ on expressions $\Theta \vdash t : \mathtt{R}$ which naturally corresponds to functions from $\mathbb{R}^n$ to $\mathbb{R}$, for which belonging to $\mathfrak{F}$ is indeed meaningful.

**Definition 1 (Open Logical Predicate).** *Let $\Theta = x_1 : \mathtt{R}, \ldots, x_n : \mathtt{R}$ be a fixed environment. We define the type-indexed family of predicates $\mathcal{F}_\tau^\Theta$ by induction on $\tau$ as follows:*

$$\begin{aligned} t \in \mathcal{F}_\mathtt{R}^\Theta &\iff (\Theta \vdash t : \mathtt{R} \wedge [\![\Theta \vdash t : \mathtt{R}]\!] \in \mathfrak{F}) \\ t \in \mathcal{F}_{\tau_1 \to \tau_2}^\Theta &\iff (\Theta \vdash t : \tau_1 \to \tau_2 \wedge \forall s \in \mathcal{F}_{\tau_1}^\Theta.\ ts \in \mathcal{F}_{\tau_2}^\Theta) \\ t \in \mathcal{F}_{\tau_1 \times \tau_2}^\Theta &\iff (\Theta \vdash t : \tau_1 \times \tau_2 \wedge \forall i \in \{1,2\}.\ t.i \in \mathcal{F}_{\tau_i}^\Theta). \end{aligned}$$

*We extend* $\mathcal{F}_\tau^\Theta$ *to the predicate* $\mathcal{F}_\tau^{\Gamma,\Theta}$*, where* $\Gamma$ *ranges over arbitrary environments (possibly containing variables of type* $\mathtt{R}$*) as follows:*

$$t \in \mathcal{F}_\tau^{\Gamma,\Theta} \iff (\Gamma, \Theta \vdash t : \tau \wedge \forall \gamma.\ \gamma \in \mathcal{F}_\Theta^\Gamma \implies t\gamma \in \mathcal{F}_\tau^\Theta).$$

*Here,* $\gamma$ *ranges over substitutions*[6] *and* $\gamma \in \mathcal{F}_\Theta^\Gamma$ *holds if the support of* $\gamma$ *is* $\Gamma$ *and* $\gamma(x) \in \mathcal{F}_\tau^\Theta$*, for any* $(x : \tau) \in \Gamma$*.*

Notice that Definition 1 ensures first-order real-valued functions to be in $\mathfrak{F}$, and asks for such a property to be hereditary preserved at higher-order types. Lemma 1 states that these conditions are indeed sufficient to guarantee any $\Lambda_{\mathfrak{F}}^{\times,\to,\mathtt{R}}$ term $\Theta \vdash t : \mathtt{R}$ to denote a function in $\mathfrak{F}$.

**Lemma 1 (Fundamental Lemma).** *For all environments* $\Gamma, \Theta$ *as above, and for any expression* $\Gamma, \Theta \vdash t : \tau$*, we have* $t \in \mathcal{F}_\tau^{\Gamma,\Theta}$*.*

*Proof.* By induction on $t$, observing that $\mathcal{F}_\tau^\Theta$ is closed under denotational semantics: if $s \in \mathcal{F}_\tau^\Theta$ and $[\![\Theta \vdash t : \tau]\!] = [\![\Theta \vdash s : \tau]\!]$, then $t \in \mathcal{F}_\tau^\Theta$. The proof follows the same structure of Lemma 3, and thus we omit details here. □

Finally, a straightforward application of Lemma 1 gives the desired result, namely Theorem 1.

## 5 Automatic Differentiation

In this section, we show how we can use open logical relations to prove the correctness of (a fragment of) the automatic differentiation algorithm of [50] (suitably adapted to our calculus).

*Automatic differentiation* [8,9,35] (AD, for short) is a family of techniques to efficiently compute the *numerical* (as opposed to *symbolical*) derivative of a computer program denoting a real-valued function. Roughly speaking, AD acts on the code of a program by letting variables incorporate values for their derivative, and operators propagate derivatives according to the *chain rule* of differential calculus [52]. Due to its vast applications in machine learning (backpropagation [49] being an example of an AD technique) and, most notably, in deep learning [9], AD is rapidly becoming a topic of interest in the programming language theory community, as witnessed by the new line of research called *differentiable programming* (see, e.g., [28,50,16,1] for some recent results on AD and programming language theory developed in the latter field).

AD comes several modes, the two most important ones being the *forward mode* (also called *tangent mode*) and the *backward mode* (also called *reverse mode*). These can be seen as different ways to compute the chain rule, the former by traversing the chain rule from inside to outside, while the latter from outside to inside.

[6] We write $t\gamma$ for the result of applying $\gamma$ to variables in $t$.

Here we are concerned with forward mode AD. More specifically, we consider the forward mode AD algorithm recently proposed in [50]. The latter is based on a source-to-source program transformation extracting out of a program $t$ a new program $\mathtt{D}t$ whose evaluation simultaneously gives the result of computing $t$ and its derivative. This is achieved by augmenting the code of $t$ in such a way to handle *dual numbers*[7].

The transformation roughly goes as follows: expressions $s$ of type $\mathtt{R}$ are transformed into dual numbers, i.e. expressions $s'$ of type $\mathtt{R} \times \mathtt{R}$, where the first component of $s'$ gives the original value of $s$, and the second component of $s'$ gives the derivative of $s$. Real-valued function symbols are then extended to handle dual numbers by applying the chain rule, while other constructors of the language are extended pointwise.

The algorithm of [50] has been studied by means of benchmarks and, to the best of the authors' knowledge, the only proof of its correctness available in the literature[8] has been given at the time of writing by Huot et al. in [37]. However, the latter proof relies on *denotational* semantics, and no *operational* proof of correctness has been given so far. Differentiability being a first-order concept, open logical relations are thus a perfect candidate for such a job.

*An AD Program Transformation* In the rest of this section, given a differentiable function $f : \mathbb{R}^n \to \mathbb{R}$, we denote by $\partial_x f : \mathbb{R}^n \to \mathbb{R}$ its partial derivative with respect to the variable $x$. Let $\mathfrak{D}$ be the collection of (real-valued) differentiable functions, and let us fix a collection $\mathfrak{F}$ of real-valued functions such that, for any $f \in \mathfrak{D}$, both $f$ and $\partial_x f$ belong to $\mathfrak{F}$. We also assume $\mathfrak{F}$ to contain functions for real number arithmetic. Notice that since $\partial_x f$ is not necessarily differentiable, in general $\partial_x f \notin \mathfrak{D}$.

We begin by recalling how the program transformation of [50] works on $\Lambda_{\mathfrak{D}}^{\times,\to,\mathtt{R}}$, the extension of $\Lambda^{\times,\to,\mathtt{R}}$ with operators for functions in $\mathfrak{D}$. In order to define the derivative of a $\Lambda_{\mathfrak{D}}^{\times,\to,\mathtt{R}}$ expression, we first define an intermediate *program transformation* $\mathtt{D} : \Lambda_{\mathfrak{D}}^{\times,\to,\mathtt{R}} \to \Lambda_{\mathfrak{F}}^{\times,\to,\mathtt{R}}$ such that:

$$\Gamma \vdash t : \tau \implies \mathtt{D}\Gamma \vdash \mathtt{D}t : \mathtt{D}\tau.$$

The action of $\mathtt{D}$ on types, environments, and expressions is defined in Figure 2. Notice that $t$ is an expression in $\Lambda_{\mathfrak{D}}^{\times,\to,\mathtt{R}}$, whereas $\mathtt{D}t$ is an expression in $\Lambda_{\mathfrak{F}}^{\times,\to,\mathtt{R}}$.

Let us comment the definition of $\mathtt{D}$, beginning with its action on types. Following the rationale behind forward-mode AD, the map $\mathtt{D}$ associates to the type

[7] We represent dual numbers [21] as pairs of the form $(x, x')$, with $x, x' \in \mathbb{R}$. The first component, namely $x$, is subject to the usual real number arithmetic, whereas the second component, namely $x'$, obeys to first-order differentiation arithmetic. Dual numbers are usually presented, in analogy with complex numbers, as formal sums of the form $x + x'\varepsilon$, where $\varepsilon$ is an abstract number (an infinitesimal) subject to the law $\varepsilon^2 = 0$.

[8] However, we remark that formal approaches to *backward* automatic differentiation for higher-order languages have been recently proposed in [1,16] (see Section 7).

$$\mathtt{D}\mathtt{R} = \mathtt{R} \times \mathtt{R} \qquad \mathtt{D}(\cdot) = \cdot$$

$$\mathtt{D}(\tau_1 \times \tau_2) = \mathtt{D}\tau_1 \times \mathtt{D}\tau_2 \qquad \mathtt{D}(x : \tau, \Gamma) = \mathtt{d}x : \mathtt{D}\tau, \mathtt{D}\Gamma$$

$$\mathtt{D}(\tau_1 \to \tau_2) = \mathtt{D}\tau_1 \to \mathtt{D}\tau_2$$

$$\mathtt{D}\underline{r} = (\underline{r}, \underline{0}) \quad \mathtt{D}(\underline{f}(t_1, \dots, t_n)) = (\underline{f}(\mathtt{D}t_1.1, \dots, \mathtt{D}t_n.1), \sum_{i=1}^{n} \underline{\partial_{x_i} f}(\mathtt{D}t_1.1, \dots, \mathtt{D}t_n.1) * \mathtt{D}t_i.2)$$

$$\mathtt{D}x = \mathtt{d}x \quad \mathtt{D}(\lambda x.t) = \lambda \mathtt{d}x.\mathtt{D}t \quad \mathtt{D}(st) = (\mathtt{D}s)(\mathtt{D}t) \quad \mathtt{D}(t.i) = \mathtt{D}t.i \quad \mathtt{D}(t_1, t_2) = (\mathtt{D}t_1, \mathtt{D}t_2)$$

Fig. 2: Intermediate transformation D

R the product type $\mathtt{R} \times \mathtt{R}$, the first and second components of its inhabitants being the original expression and its derivative, respectively. The action of D on non-basic types is straightforward and it is designed so that the automatic differentiation machinery can handle higher-order expressions in such a way to guarantee correctness at real-valued function types.

The action of D on the usual constructors of the $\lambda$-calculus is pointwise, although it is worth noticing that D associates to any variable $x$ of type $\tau$ a new variable, which we denote by $\mathtt{d}x$, of type $\mathtt{D}\tau$. As we are going to see, if $\tau = \mathtt{R}$, then $\mathtt{d}x$ acts as a placeholder for a dual number.

More interesting is the action of D on real-valued constructors. To any numeral $\underline{r}$, D associates the pair $\mathtt{D}\underline{r} = (\underline{r}, \underline{0})$, the derivative of a number being zero. Let us now inspect the action of D on an operator $\underline{f}$ associated to $f : \mathbb{R}^n \to \mathbb{R}$ (we treat $f$ as a function in the variables $x_1, \dots, x_n$). The interesting part is the second component of $\mathtt{D}(\underline{f}(t_1, \dots, t_n))$, namely

$$\sum_{i=1}^{n} \underline{\partial_{x_i} f}(\mathtt{D}t_1.1, \dots, \mathtt{D}t_n.1) * \mathtt{D}t_i.2$$

where $\sum_{i=1}^{n}$ and $*$ denote the operators (of $\Lambda_{\mathfrak{F}}^{\times,\to,\mathtt{R}}$) associated to summation and (binary) multiplication (for readability we omit the underline notation), and $\underline{\partial_{x_i} f}$ is the operator (of $\Lambda_{\mathfrak{F}}^{\times,\to,\mathtt{R}}$) associated to partial derivative $\partial_{x_i} f$ of $f$ in the variable $x_i$. It is not hard to recognize that the above expression is nothing but an instance of the *chain rule*.

Finally, we notice that if $\Gamma \vdash t : \tau$ is a (derivable) judgment in $\Lambda_{\mathfrak{D}}^{\times,\to,\mathtt{R}}$, then indeed $\mathtt{D}\Gamma \vdash \mathtt{D}t : \mathtt{D}\tau$ is a (derivable) judgment in $\Lambda_{\mathfrak{F}}^{\times,\to,\mathtt{R}}$.

*Example 1.* Let us consider the binary function $f(x_1, x_2) = \sin(x_1) + \cos(x_2)$. For readability, we overload the notation writing $f$ in place of $\underline{f}$ (and similarly for $\partial_{x_i} f$). Given expressions $t_1, t_2$, we compute $\mathtt{D}(\sin(t_1) + \cos(t_2))$. Recall that

$\partial_{x_1} f(x_1, x_2) = \cos(x_1)$ and $\partial_{x_2} f(x_1, x_2) = -\sin(x_2)$. We have:

$$\begin{aligned}
&\mathtt{D}(\sin(t_1) + \cos(t_2)) \\
&= (\sin(\mathtt{D}t_1.1) + \cos(\mathtt{D}t_2.1), \partial_{x_1} f(\mathtt{D}t_1.1, \mathtt{D}t_2.1) * \mathtt{D}t_1.2 + \partial_{x_2} f(\mathtt{D}t_1.1, \mathtt{D}t_2.1) * \mathtt{D}t_2.2) \\
&= (\sin(\mathtt{D}t_1.1) + \cos(\mathtt{D}t_2.1), \cos(\mathtt{D}t_1.1) * \mathtt{D}t_1.2 - \sin(\mathtt{D}t_2.1) * \mathtt{D}t_2.2).
\end{aligned}$$

As a consequence, we see that $\mathtt{D}(\lambda x.\lambda y. \sin(x) + \cos(y))$ is

$$\lambda \mathtt{d}x.\lambda \mathtt{d}y.(\sin(\mathtt{d}x.1) + \cos(\mathtt{d}y.1), \cos(\mathtt{d}x.1) * \mathtt{d}x.2 - \sin(\mathtt{d}y.1) * \mathtt{d}y.2).$$

We now aim to define the derivative of an expression $x_1 : \mathtt{R}, \ldots, x_n : \mathtt{R} \vdash t : \mathtt{R}$ with respect to a variable $x$ (of type $\mathtt{R}$). In order to do so we first associate to any variable $y : \mathtt{R}$ its dual expression $\mathtt{dual}_x(y) : \mathtt{R} \times \mathtt{R}$ defined as:

$$\mathtt{dual}_x(y) = \begin{cases} (y, \underline{1}) & \text{if } x = y \\ (y, \underline{0}) & \text{otherwise.} \end{cases}$$

Next, we define for $x_1 : \mathtt{R}, \ldots, x_n : \mathtt{R} \vdash t : \mathtt{R}$ the derivative $\mathtt{deriv}(x, t)$ of $t$ with respect to $x$ as:

$$\mathtt{deriv}(x, t) = \mathtt{D}t[\mathtt{dual}_x(x_1)/\mathtt{d}x_1, \ldots, \mathtt{dual}_x(x_n)/\mathtt{d}x_n].2$$

Let us clarify this passage with a simple example.

*Example 2.* Let us compute the derivative of $x : \mathtt{R}, y : \mathtt{R} \vdash t : \mathtt{R}$, where $t = x * y$. We first of all compute $\mathtt{D}t$, obtaining:

$$\mathtt{d}x : \mathtt{R} \times \mathtt{R}, \mathtt{d}y : \mathtt{R} \times \mathtt{R} \vdash ((\mathtt{d}x.1) * (\mathtt{d}y.1), (\mathtt{d}x.1) * (\mathtt{d}y.2) + (\mathtt{d}x.2) * (\mathtt{d}y.1)) : \mathtt{R} \times \mathtt{R}.$$

Observing that $\mathtt{dual}_x(x) = (x, \underline{1})$ and $\mathtt{dual}_x(y) = (y, \underline{0})$, we indeed obtain the desired derivative as $x : \mathtt{R}, y : \mathtt{R} \vdash \mathtt{D}t[\mathtt{dual}_x(x)/\mathtt{d}x, \mathtt{dual}_x(y)/\mathtt{d}y].2 : \mathtt{R}$. For we have:

$$\begin{aligned}
&[\![x : \mathtt{R}, y : \mathtt{R} \vdash \mathtt{D}t[\mathtt{dual}_x(x)/\mathtt{d}x, \mathtt{dual}_x(y)/\mathtt{d}y].2 : \mathtt{R}]\!] \\
&= [\![x : \mathtt{R}, y : \mathtt{R} \vdash (x * y, x * 0 + 1 * y).2 : \mathtt{R}]\!] \\
&= [\![x : \mathtt{R}, y : \mathtt{R} \vdash y : \mathtt{R}]\!] = \partial_x [\![x : \mathtt{R}, y : \mathtt{R} \vdash x * y : \mathtt{R}]\!].
\end{aligned}$$

*Remark 1.* For $\Theta = x_1 : \mathtt{R}, \ldots, x_n : \mathtt{R}$ we have $\Theta \vdash \mathtt{dual}_y(x_i) : \mathtt{DR}$ and $\Theta \vdash \mathtt{D}s[\mathtt{dual}_y(x_1)/\mathtt{d}x_1, \ldots, \mathtt{dual}_y(x_n)/\mathtt{d}x_n] : \mathtt{D}\tau$, for any variable $y$ and $\Theta \vdash s : \tau$.

*Open Logical relations for AD* We have claimed that the operation $\mathtt{deriv}$ performs automatic differentiation of $\Lambda_{\mathfrak{D}}^{\times,\to,\mathtt{R}}$ expressions. By that we mean that once applied to expressions of the form $x_1 : \mathtt{R}, \ldots, x_n : \mathtt{R} \vdash t : \mathtt{R}$, the operation $\mathtt{deriv}$ can be used to compute the derivative of $[\![x_1 : \mathtt{R}, \ldots, x_n : \mathtt{R} \vdash t : \mathtt{R}]\!]$. We now show how we can prove such a statement using open logical relations, this way providing a proof of correctness of our AD program transformation.

We begin by defining a logical relations $\mathcal{R}$ between $\Lambda_{\mathfrak{D}}^{\times,\to,\mathtt{R}}$ and $\Lambda_{\mathfrak{F}}^{\times,\to,\mathtt{R}}$ expressions. We design $\mathcal{R}$ in such a way that (i) $t \mathcal{R} \mathtt{D}t$ and (ii) if $t \mathcal{R} s$ and $t$ inhabits a first-order type, then indeed $s$ corresponds to the derivative of $t$. While (ii) essentially holds by definition, (i) requires some efforts in order to be proved.

**Definition 2 (Open Logical Relation).** *Let $\Theta = x_1 : \mathtt{R}, \dots, x_n : \mathtt{R}$ be a fixed, arbitrary environment. Define the family of relations $(\mathcal{R}^{\Theta}_{\tau})_{\Theta,\tau}$ between $\Lambda^{\times,\to,\mathtt{R}}_{\mathfrak{D}}$ and $\Lambda^{\times,\to,\mathtt{R}}_{\mathfrak{F}}$ expressions by induction on $\tau$ as follows:*

$$t\,\mathcal{R}^{\Theta}_{\mathtt{R}}\,s \iff \begin{cases} \Theta \vdash t : \mathtt{R} \wedge \mathtt{D}\Theta \vdash s : \mathtt{R} \times \mathtt{R} \\ \forall y : \mathtt{R}. \\ [\![\Theta \vdash s[\mathtt{dual}_y(x_1)/\mathtt{d}x_1, \dots, \mathtt{dual}_y(x_n)/\mathtt{d}x_n].1 : \mathtt{R}]\!] = [\![\Theta \vdash t : \mathtt{R}]\!] \\ [\![\Theta \vdash s[\mathtt{dual}_y(x_1)/\mathtt{d}x_1, \dots, \mathtt{dual}_y(x_n)/\mathtt{d}x_n].2 : \mathtt{R}]\!] = \partial_y [\![\Theta \vdash t : \mathtt{R}]\!] \end{cases}$$

$$t\,\mathcal{R}^{\Theta}_{\tau_1 \to \tau_2}\,s \iff \begin{cases} \Theta \vdash t : \tau_1 \to \tau_2 \wedge \mathtt{D}\Theta \vdash s : \mathtt{D}\tau_1 \to \mathtt{D}\tau_2 \\ \forall p, q.\ p\,\mathcal{R}^{\Theta}_{\tau_1}\,q \implies tp\,\mathcal{R}^{\Theta}_{\tau_2}\,sq \end{cases}$$

$$t\,\mathcal{R}^{\Theta}_{\tau_1 \times \tau_2}\,s \iff \begin{cases} \Theta \vdash t : \tau_1 \times \tau_2 \wedge \mathtt{D}\Theta \vdash s : \mathtt{D}\tau_1 \times \mathtt{D}\tau_2 \\ \forall i \in \{1, 2\}.\ t.i\,\mathcal{R}^{\Theta}_{\tau_i}\,s.i \end{cases}$$

*We extend $\mathcal{R}^{\Theta}_{\tau}$ to the family $(\mathcal{R}^{\Gamma,\Theta}_{\tau})_{\Gamma,\Theta,\tau}$, where $\Gamma$ ranges over arbitrary environments (possibly containing variables of type $\mathtt{R}$), as follows:*

$$t\,\mathcal{R}^{\Gamma,\Theta}_{\tau}\,s \iff (\Gamma, \Theta \vdash t : \tau) \wedge (\mathtt{D}\Gamma, \mathtt{D}\Theta \vdash s : \mathtt{D}\tau) \wedge (\forall \gamma, \delta.\ \gamma\,\mathcal{R}^{\Gamma}_{\Theta}\,\delta \implies t\gamma\,\mathcal{R}^{\Theta}_{\tau}\,s\delta)$$

*where $\gamma$, $\delta$ range over substitutions, and:*

$$\gamma\,\mathcal{R}^{\Gamma}_{\Theta}\,\delta \iff (\mathsf{supp}(\gamma) = \Gamma) \wedge (\mathsf{supp}(\delta) = \mathtt{D}\Gamma) \wedge (\forall (x : \tau) \in \Gamma.\ \gamma(x)\,\mathcal{R}^{\Theta}_{\tau}\,\delta(\mathtt{d}x)).$$

Obviously, Definition 2 satisfies condition (ii) above. What remains to be done is to show that it satisfies condition (i) as well. In order to prove such a result, we first need to show that the logical relation respects the denotational semantics of $\Lambda^{\times,\to,\mathtt{R}}_{\mathfrak{D}}$.

**Lemma 2.** *Let $\Theta = x_1 : \mathtt{R}, \dots, x_n : \mathtt{R}$. Then, the following hold:*

$$t'\,\mathcal{R}^{\Theta}_{\tau}\,s \wedge [\![\Theta \vdash t : \tau]\!] = [\![\Theta \vdash t' : \tau]\!] \implies t\,\mathcal{R}^{\Theta}_{\tau}\,s$$
$$t\,\mathcal{R}^{\Theta}_{\tau}\,s' \wedge [\![\mathtt{D}\Theta \vdash s' : \mathtt{D}\tau]\!] = [\![\mathtt{D}\Theta \vdash s : \mathtt{D}\tau]\!] \implies t\,\mathcal{R}^{\Theta}_{\tau}\,s.$$

*Proof.* A standard induction on $\tau$. □

We are now ready to state and prove the main result of this section.

**Lemma 3 (Fundamental Lemma).** *For all environments $\Gamma, \Theta$ and for any expression $\Gamma, \Theta \vdash t : \tau$, we have $t\,\mathcal{R}^{\Gamma,\Theta}_{\tau}\,\mathtt{D}t$.*

*Proof.* We prove the following statement, by induction on $t$:

$$\forall t.\ \forall \tau.\ \forall \Gamma, \Theta.\ (\Gamma, \Theta \vdash t : \tau \implies t\,\mathcal{R}^{\Gamma,\Theta}_{\tau}\,\mathtt{D}t).$$

We show only the most relevant cases. Suppose $t$ is a variable $x$. We distinguish whether $x$ belongs to $\Gamma$ or $\Theta$.

1. Suppose $(x : \mathtt{R}) \in \Theta$. We have to show $x\, \mathcal{R}_{\mathtt{R}}^{\Gamma,\Theta}\, \mathtt{d}x$, i.e.

$$\llbracket \Theta \vdash \mathtt{d}x[\mathtt{dual}_y(x)/\mathtt{d}x].1 : \mathtt{R} \rrbracket = \llbracket \Theta \vdash x : \mathtt{R} \rrbracket$$
$$\llbracket \Theta \vdash \mathtt{d}x[\mathtt{dual}_y(x)/\mathtt{d}x].2 : \mathtt{R} \rrbracket = \partial_y \llbracket \Theta \vdash x : \mathtt{R} \rrbracket$$

for any variable $y$ (of type $\mathtt{R}$). The first identity obviously holds as

$$\llbracket \Theta \vdash \mathtt{d}x[\mathtt{dual}_y(x)/\mathtt{d}x].1 : \mathtt{R} \rrbracket = \llbracket \Theta \vdash \mathtt{d}x[(x, b)/\mathtt{d}x].1 : \mathtt{R} \rrbracket = \llbracket \Theta \vdash x : \mathtt{R} \rrbracket,$$

where $b \in \{\underline{0}, \underline{1}\}$. For the second identity we distinguish whether $y = x$ or $y \neq x$. In the former case we have $\mathtt{dual}_y(x) = (x, \underline{1})$, and thus:

$$\llbracket \Theta \vdash \mathtt{d}x[\mathtt{dual}_y(x)/\mathtt{d}x].2 : \mathtt{R} \rrbracket = \llbracket \Theta \vdash \underline{1} : \mathtt{R} \rrbracket = \partial_y \llbracket \Theta \vdash y : \mathtt{R} \rrbracket.$$

In the latter case we have $\mathtt{dual}_y(x) = (x, \underline{0})$, and thus:

$$\llbracket \Theta \vdash \mathtt{d}x[\mathtt{dual}_y(x)/\mathtt{d}x].2 : \mathtt{R} \rrbracket = \llbracket \Theta \vdash \underline{0} : \mathtt{R} \rrbracket = \partial_y \llbracket \Theta \vdash x : \mathtt{R} \rrbracket.$$

2. Suppose $(x : \tau) \in \Gamma$. We have to show $x\, \mathcal{R}^{\Gamma,\Theta}\, \mathtt{d}x$, i.e. $\gamma(x)\, \mathcal{R}_\tau^\Theta\, \delta(\mathtt{d}x)$, for all substitutions $\gamma, \delta$ such that $\gamma\, \mathcal{R}_\Theta^\Gamma\, \delta$. Since $x$ belongs to $\Gamma$, we are trivially done.

Suppose $t$ is $\lambda x.s$, so that we have

$$\frac{\Gamma, \Theta, x : \tau_1 \vdash s : \tau_2}{\Gamma, \Theta \vdash \lambda x.s : \tau_1 \to \tau_2}$$

for some types $\tau_1, \tau_2$. As $x$ is bound in $\lambda x.s$, without loss of generality we can assume $(x : \tau_1) \notin \Gamma \cup \Theta$. Let $\Delta = \Gamma, x : \tau_1$, so that we have $\Delta, \Theta \vdash s : \tau_2$, and thus $s\, \mathcal{R}_{\tau_2}^{\Delta,\Theta}\, \mathtt{D}s$, by induction hypothesis. By definition of open logical relation, we have to prove that for arbitrary $\gamma, \delta$ such that $\gamma\, \mathcal{R}_\Theta^\Gamma\, \delta$, we have

$$\lambda x.s\gamma\, \mathcal{R}_{\tau_1 \to \tau_2}^\Theta\, \lambda \mathtt{d}x.(\mathtt{D}s)\delta,$$

i.e. $(\lambda x.s\gamma)p\, \mathcal{R}_{\tau_2}^\Theta\, (\lambda \mathtt{d}x.(\mathtt{D}s)\delta)q$, for all $p\, \mathcal{R}_{\tau_1}^\Theta\, q$. Let us fix a pair $(p, q)$ as above. By Lemma 2, it is sufficient to show $(s\gamma)[p/x]\, \mathcal{R}_{\tau_2}^\Theta\, ((\mathtt{D}s)\delta)[q/\mathtt{d}x]$. Let $\gamma', \delta'$ be the substitutions defined as follows:

$$\gamma'(y) = \begin{cases} p & \text{if } y = x \\ \gamma(y) & \text{otherwise} \end{cases} \qquad \delta'(y) = \begin{cases} q & \text{if } y = \mathtt{d}x \\ \delta(y) & \text{otherwise.} \end{cases}$$

It is easy to see that $\gamma'\, \mathcal{R}_\Theta^\Delta\, \delta'$, so that by $s\, \mathcal{R}_{\tau_2}^{\Delta,\Theta}\, \mathtt{D}s$ (recall that the latter follows by induction hypothesis) we infer $s\gamma'\, \mathcal{R}_{\tau_2}^\Theta\, (\mathtt{D}s)\delta'$, by the very definition of open logical relation. As a consequence, the thesis is proved if we show

$$(s\gamma)[p/x] = s\gamma'; \qquad ((\mathtt{D}s)\delta)[q/\mathtt{d}x] = (\mathtt{D}s)\delta'.$$

The above identities hold if $x \notin FV(\gamma(y))$ and $\mathtt{d}x \notin FV(\delta(\mathtt{d}y))$, for any $(y : \tau) \in \Gamma$. This is indeed the case, since $\gamma(y)\, \mathcal{R}_\tau^\Theta\, \delta(\mathtt{d}y)$ implies $\Theta \vdash \gamma(y) : \tau$ and $\mathtt{D}\Theta \vdash \delta(\mathtt{d}y) : \mathtt{D}\tau$, and $x \notin \Theta$ (and thus $\mathtt{d}x \notin \mathtt{D}\Theta$). $\square$

A direct application of Lemma 3 allows us to conclude the correctness of the program transformation D. In fact, given a first-order term $\Theta \vdash t : \mathtt{R}$, with $\Theta = x_1 : \mathtt{R}, \ldots, x_n : \mathtt{R}$, by Lemma 3 we have $t \, \mathcal{R}^{\Theta}_{\mathtt{R}} \, \mathtt{D}t$, and thus

$$\partial_y [\![\Theta \vdash t : \mathtt{R}]\!] = [\![\Theta \vdash \mathtt{D}t[\mathtt{dual}_y(x_1)/\mathtt{d}x_1, \ldots, \mathtt{dual}_y(x_n)/\mathtt{d}x_n].2 : \mathtt{R}]\!],$$

for any real-valued variable $y$, meaning that $\mathtt{D}t$ indeed computes the partial derivative of $t$.

**Theorem 2.** *For any term $\Theta \vdash t : \mathtt{R}$ as above, the term $\mathtt{D}\Theta \vdash \mathtt{D}t : \mathtt{DR}$ computes the partial derivative of $t$, i.e., for any variable $y$ we have*

$$\partial_y [\![\Theta \vdash t : \mathtt{R}]\!] = [\![\Theta \vdash \mathtt{D}t[\mathtt{dual}_y(x_1)/\mathtt{d}x_1, \ldots, \mathtt{dual}_y(x_n)/\mathtt{d}x_n].2 : \mathtt{R}]\!].$$

## 6 On Refinement Types and Local Continuity

In Section 4, we exploited open logical relations to establish a containment theorem for the calculus $\Lambda^{\times,\rightarrow,\mathtt{R}}_{\mathfrak{F}}$, i.e. the calculus $\Lambda^{\times,\rightarrow,\mathtt{R}}$ extended with real-valued functions belonging to a set $\mathfrak{F}$ including projections and closed under function composition. Since the collection $\mathfrak{C}$ of (real-valued) *continuous* functions satisfies both constraints, Theorem 1 allows us to conclude that all first order terms of $\Lambda^{\times,\rightarrow,\mathtt{R}}_{\mathfrak{C}}$ represent continuous functions.

The aim of the present section is the development of a framework to prove continuity properties of programs in a calculus that goes *beyond* $\Lambda^{\times,\rightarrow,\mathtt{R}}_{\mathfrak{C}}$. More specifically, (i) we do not restrict our analysis to calculi having operators representing continuous real-valued functions only, but consider operators for arbitrary real-valued functions, and (ii) we add to our calculus an if-then-else construct whose static semantics is captured by the following rule:

$$\frac{\Gamma \vdash t : \mathtt{R} \quad \Gamma \vdash s : \tau \quad \Gamma \vdash p : \tau}{\Gamma \vdash \mathtt{if}\ t\ \mathtt{then}\ s\ \mathtt{else}\ p : \tau}$$

The intended dynamic semantics of the term $\mathtt{if}\ t\ \mathtt{then}\ s\ \mathtt{else}\ p$ is the same as the one of $s$ whenever $t$ evaluates to any real number $r \neq 0$ and the same as the one of $p$ if it evaluates to 0.

Notice that the crux of the problem we aim to solve is the presence of the if-then-else construct. Indeed, independently of point (i), such a construct breaks the global continuity of programs, as illustrated in Figure 3a. As a consequence we are forced to look at *local* continuity properties, instead: for instance we can say that the program of Figure 3a is continuous both on $\mathbb{R}_{<0}$ and $\mathbb{R}_{\geq 0}$. Observe that guaranteeing local continuity allows us (up to a certain point) to recover the ability of approximating the output of a program by approximating its input. Indeed, if a program $t : \mathtt{R} \times \ldots \times \mathtt{R} \rightarrow \mathtt{R}$ is *locally continuous* on a subset $X$ of $\mathbb{R}^n$, then the value of $ts$ (for some input $s$) can be approximated

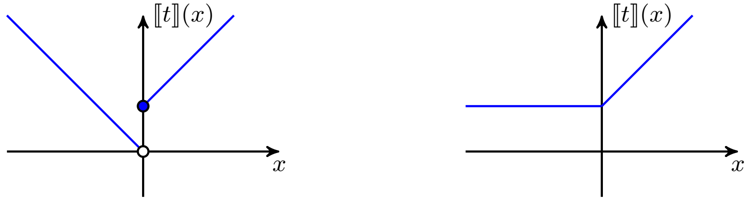

(a) $t = \lambda x.\texttt{if}\ x < 0\ \texttt{then}\ -x\ \texttt{else}\ x+1$ (b) $t = \lambda x.\texttt{if}\ x < 0\ \texttt{then}\ 1\ \texttt{else}\ x+1$

Fig. 3: Simply typed first-order programs with branches by passing as argument to $t$ a family $(s_n)_{n\in\mathbb{N}}$ of approximations of $s$, *as long as* both $s$ and all the $(s_n)_{n\in\mathbb{N}}$ are indeed elements of $X$. Notice that the continuity domains we are interested in are not necessary open sets: we could for instance be interested in functions that are continuous on the unit circle, i.e. the points $\{(a, b) \mid a^2 + b^2 = 1\} \subseteq \mathbb{R}^2$. For this reason we will work with the notion of *sequential* continuity, instead of the usual topological notion of continuity. It must be observed, however, that these two notions coincide as soon as the continuity domain $X$ is actually an open set.

**Definition 3 (Sequential Continuity).** *Let $f : \mathbb{R}^n \to \mathbb{R}$, and $X$ be any subset of $\mathbb{R}^n$. We say that $f$ is* (sequentially) continuous *on $X$ if for every $x \in X$, and for every sequence $(x_n)_{n\in\mathbb{N}}$ of elements of $X$ such that* $\lim_{n\to\infty} x_n = x$*, it holds that* $\lim_{n\to\infty} f(x_n) = f(x)$.

In [18], Chaudhuri et al. introduced a logical system designed to guarantee local continuity properties on programs in an *imperative* (first-order) programming language with conditional branches and loops. In this section, we develop a similar system in the setting of a *higher-order functional language* with an if-then-else construct, and we use open logical relations to prove the soundness of our system. This witnesses, on yet another situation, the versatility of open logical relations. Compared to [18], we somehow generalize from a result on programs built from only first-order constructs and primitive functions, to a containment result for programs built using also higher-order constructs.

We however mention that, although our system is inspired by the work of Chaudhuri at al., there are significant differences between the two, even at the first-order level. The consequences these differences have on the expressive power of our systems are twofold:

- On the one hand, while inferring continuity on some domain $X$ of a program of the form $\texttt{if}\ t\ \texttt{then}\ s\ \texttt{else}\ p$, we have more flexibility than [18] for the domains of continuity of $s$ and $p$. To be more concrete, let us consider the program $\lambda x.(\texttt{if}\ (x > 0)\ \texttt{then}\ 0\ \texttt{else}\ (\texttt{if}\ x = 4\ \texttt{then}\ 1\ \texttt{else}\ 0))$, which is continuous on $\mathbb{R}$ even though the second branch is continuous on $\mathbb{R}_{\leq 0}$, but not on $\mathbb{R}$. We are able to show in our system that this program is indeed continuous on *the whole* domain $\mathbb{R}$, while Chaudhuri et al. cannot do the same in their system for the corresponding imperative program: they ask the domain of continuity of *each* of the two branches to *coincide* with the domain of continuity of the whole program.

- On the other hand, the system of Chaudhuri at al. allows one to express continuity along a restricted set of variables, which we cannot do. To illustrate this, let us look at the program: $\lambda x, y.\mathtt{if}\ (x = 0)\ \mathtt{then}\ (3 * y)\ \mathtt{else}\ (4 * y)$: along the variable $y$, this program is continuous on the whole of $\mathbb{R}$. Chaudhuri et al. are able to express and prove this statement in their system, while we can only say that for every real $a$, this program is continuous on the domain $\{a\} \times \mathbb{R}$.

For the sake of simplicity, it is useful to slightly simplify our calculus; the ideas we present here, however, would still be valid in a more general setting, but that would make the presentation and proofs more involved. As usual, let $\mathfrak{F}$ be a collection of real-valued functions. We consider the restriction of the calculus $\Lambda_{\mathfrak{F}}^{\times,\to,\mathtt{R}}$ obtained by considering types of the form

$$\tau \ ::= \ \mathtt{R} \mid \rho; \qquad\qquad \rho \ ::= \ \rho_1 \times \cdots \times \rho_n \times \underbrace{\mathtt{R} \times \cdots \times \mathtt{R}}_{m\text{-times}} \to \tau;$$

only. For the sake of readability, we employ the notation $(\rho_1 \ldots, \rho_n, \mathtt{R}, \ldots, \mathtt{R}) \to \tau$ in place of $\rho_1 \times \cdots \times \rho_n \times \mathtt{R} \times \cdots \times \mathtt{R} \to \tau$. We also overload the notation and keep indicating the resulting calculus as $\Lambda_{\mathfrak{F}}^{\times,\to,\mathtt{R}}$. Nonetheless, the reader should keep in mind that from now on, whenever referring to a $\Lambda_{\mathfrak{F}}^{\times,\to,\mathtt{R}}$ term, we are tacitly referring to a term typable according to the restricted type system, but that can indeed contain conditionals.

Since we want to be able to talk about *composition properties* of locally continuous programs, we actually need to talk not only about the points where a program is continuous, but also about the *image* of this continuity domain. In higher-order languages, a well-established framework for the latter kind of specifications is the one of *refinement types*, that have been first introduced by [31] in the context of ML types: the basic idea is to annotate an existing type system with logical formulas, with the aim of being more precise about the underlying program's behaviors than in simple types. Here, we are going to adapt this framework by replacing the image annotations provided by standard refinement types with *continuity annotations.*

### 6.1 A Refinement Type System Ensuring Local Continuity

Our refinement type system is developed on top of the simple types system of Section 2 (actually, on the simplification of such a system we are considering in this section). We first need to introduce a set of logical formulas which talk about $n$-uples of real numbers, and which we use as annotations in our refinement types. We consider a set $\mathcal{V}$ of logical variables, and we construct formulas as follows:

$$\psi, \phi \in \mathcal{L} \ ::= \ \top \ \Big| \ (e \leq e) \ \Big| \ \psi \wedge \phi \ \Big| \ \neg\psi,$$

$$e \in \mathcal{E} \ ::= \ \alpha \ \Big| \ a \ \Big| \ f(e, \ldots, e) \qquad \text{with } \alpha \in \mathcal{V}, a \in \mathbb{R}, f : \mathbb{R}^n \to \mathbb{R}.$$

Recall that with the connectives in our logic, we are able to encode logical disjunction and implication, and as customary, we write $\phi \Rightarrow \psi$ for $\neg\phi \vee \psi$. A *real assignment* is a partial map $\sigma : \mathcal{V} \to \mathbb{R}$. When $\sigma$ has finite support, we sometimes specify $\sigma$ by writing $(\alpha_1 \mapsto \sigma(\alpha_1), \dots, \alpha_n \mapsto \sigma(\alpha_n))$. We note $\sigma \models \phi$ when $\sigma$ is defined on the variables occurring in $\phi$, and moreover the real formula obtained when replacing along $\sigma$ the logical variables of $\phi$ is true. We write $\models \phi$ when $\sigma \models \phi$ always holds, independently on $\sigma$.

We can associate to every formula the subset of $\mathbb{R}^n$ consisting of all points where this formula holds: more precisely, if $\phi$ is a formula, and $X = \alpha_1, \dots, \alpha_n$ is a list of logical variables such that $\mathrm{Vars}(\phi) \subseteq X$, we call *truth domain of* $\phi$ *w.r.t.* $X$ the set:

$$\mathrm{Dom}(\phi)^X = \{(a_1, \dots, a_n) \in \mathbb{R}^n \mid (\alpha_1 \mapsto a_1, \dots, \alpha_n \mapsto a_n) \models \phi\}.$$

We are now ready to define the language of refinement types, which can be seen as simple types annotated by logical formulas. The type $\mathtt{R}$ is annotated by logical *variables*: this way we obtain *refinement real types* of the form $\{\alpha \in \mathtt{R}\}$. The crux of our refinement type system consists in the annotations we put *on the arrows*. We introduce two distinct refined arrow constructs, depending on the shape of the target type: more precisely we annotate the arrow of a type $(T_1, \dots, T_n) \to \mathtt{R}$ with *two* logical formulas, while we annotate $(T_1, \dots, T_n) \to H$ (where $H$ is an higher-order type) with only *one* logical formula. This way, we obtain refined arrow types of the form $(T_1, \dots, T_n) \overset{\psi \rightsquigarrow \phi}{\to} \{\alpha \in \mathtt{R}\}$, and $(T_1, \dots, T_n) \overset{\psi}{\to} H$: in both cases the formula $\psi$ specifies the continuity domain, while the formula $\phi$ is an *image annotation* used only when the target type is ground. The intuition is as follows: a program of type $(H_1, \dots, H_n, \{\alpha_1 \in \mathtt{R}\}, \dots, \{\alpha_n \in \mathtt{R}\}) \overset{\psi \rightsquigarrow \phi}{\to} \{\alpha \in \mathtt{R}\}$ uses its real arguments continuously on the domain specified by the formula $\psi$ (w.r.t $\alpha_1, \dots, \alpha_n$), and this domain is sent into the domain specified by the formula $\phi$ (w.r.t. $\alpha$). Similarly, a program of the type $(T_1, \dots, T_n) \overset{\psi}{\to} H$ has its real arguments used in a continuous way on the domain specified by $\psi$, but it is not possible anymore to specify an image domain, because $H$ is higher-order.

The general form of our refined types is thus as follows:

$$T ::= H \;\Big|\; F; \qquad F ::= \{\alpha \in \mathtt{R}\};$$

$$H ::= (H_1, \dots, H_m, F_1, \dots, F_n) \overset{\psi}{\to} H \;\Big|\; (H_1, \dots, H_m, F_1, \dots, F_n) \overset{\psi \rightsquigarrow \phi}{\to} F$$

with $n + m > 0$, $\mathrm{Vars}(\phi) \subseteq \{\alpha\}$, $\mathrm{Vars}(\psi) \subseteq \{\alpha_1, \dots, \alpha_n\}$ when $F = \{\alpha \in \mathtt{R}\}$, $F_i = \{\alpha_i \in \mathtt{R}\}$, and the $(\alpha_i)_{1 \le i \le n}$ are distinct. We take refinement types up to renaming of logical variables. If $T$ is a refinement type, we write $\overline{T}$ for the simple type we obtain by forgetting about the annotations in $T$.

*Example 3.* We illustrate in this example the intended meaning of our refinement types.

- We first look at how to refine $\mathtt{R} \to \mathtt{R}$: those are types of the form $\{\alpha_1 \in \mathtt{R}\} \overset{\phi_1 \rightsquigarrow \phi_2}{\to} \{\alpha_2 \in \mathtt{R}\}$. The intended inhabitants of these types are the programs

$t : \mathtt{R} \to \mathtt{R}$ such that i) $[\![t]\!]$ is continuous on the truth domain of $\phi_1$; and ii) $[\![t]\!]$ sends the truth domain of $\phi_1$ into the truth domain of $\phi_2$. As an example, $\phi_1$ could be $(\alpha_1 < 3)$, and $\phi_2$ could be $(\alpha_2 \geq 5)$. An example of a program having this type is $t = \lambda x.(\underline{5} + \underline{f}(x))$, where $f : \mathbb{R} \to \mathbb{R}$ is defined as $f(a) = \begin{cases} \frac{1}{3-a} \text{ when } a < 3 \\ 0 \text{ otherwise} \end{cases}$, and moreover we assume that $\{f, +\} \subseteq \mathfrak{F}$.

- We look now at the possible refinements of $\mathtt{R} \to (\mathtt{R} \to \mathtt{R})$: those are of the form $\{\alpha_1 \in \mathtt{R}\} \overset{\theta_1}{\to} (\{\alpha_2 \in \mathtt{R}\} \overset{\theta_2 \rightsquigarrow \theta_3}{\to} \{\alpha_3 \in \mathtt{R}\})$. The intended inhabitants of these types are the programs $t : \mathtt{R} \to (\mathtt{R} \to \mathtt{R})$ whose interpretation function $(x, y) \in \mathbb{R}^2 \mapsto [\![t]\!](x)(y)$ sends continously $\mathrm{Dom}(\theta_1)^{\alpha_1} \times \mathrm{Dom}(\theta_2)^{\alpha_2}$ into $\mathrm{Dom}(\theta_3)^{\alpha_3}$. As an example, consider $\theta_1 = (\alpha_1 < 1)$, $\theta_2 = (\alpha_2 \leq 3)$, and $\theta_3 = (\alpha_3 > 0)$. An example of a program having this type is $\lambda x_1.\lambda x_2.\underline{f}(x_1 * x_2)$ where we take $f$ as above.

A refined typing context $\Gamma$ is a list $x_1 : T_1, \ldots, x_n : T_n$, where each $T_i$ is a refinement type. In order to express continuity constraints, we need to *annotate* typing judgments by logical formulas, in a similar way as what we do for arrow types. More precisely, we consider two kinds of refined typing judgments: one for terms of ground type, and one for terms of higher-order type:

$$\Gamma \overset{\psi}{\vdash_{\mathtt{r}}} t : H; \qquad \Gamma \overset{\psi \rightsquigarrow \phi}{\vdash_{\mathtt{r}}} t : F.$$

## 6.2 Basic Typing Rules

We first consider refinement typing rules for the fragment of our language which excludes conditionals: they are given in Figure 4. We illustrate them by way of a series of examples.

*Example 4.* We first look at the typing rule var-F: if $\theta$ implies $\theta'$, then the variable $x$—that, in semantics terms, does the projection of the context $\Gamma$ to one of its component—sends continuously the truth domain of $\theta$ into the truth domain of $\theta'$. Using this rule we can, for instance, derive the following judgment:

$$x : \{\alpha \in \mathtt{R}\}, y : \{\beta \in \mathtt{R}\} \overset{(\alpha \geq 0 \wedge \beta \geq 0) \rightsquigarrow (\alpha \geq 0)}{\vdash_{\mathtt{r}}} x : \{\alpha \in \mathtt{R}\}. \quad (1)$$

*Example 5.* We now look at the Rf rule, that deals with functions from $\mathfrak{F}$. Using this rule, we can show that:

$$x : \{\alpha \in \mathtt{R}\}, y : \{\beta \in \mathtt{R}\} \overset{(\alpha \geq 0 \wedge \beta \geq 0) \rightsquigarrow (\gamma \geq 0)}{\vdash_{\mathtt{r}}} \underline{min}(x, y) : \{\gamma \in \mathtt{R}\}. \quad (2)$$

Before giving the refined typing rule for the if-then-else construct, we also illustrate on an example how the rules in Figure 4 allow us to exploit the continuity informations we have on functions in $\mathfrak{F}$, compositionally.

$$\text{var-H}\ \frac{}{\Gamma, x : H \overset{\psi}{\vdash_{\mathtt{r}}} x : H} \qquad \text{var-F}\ \frac{\models \theta \Rightarrow \theta'}{\Gamma, x : \{\alpha \in \mathtt{R}\} \overset{\theta \rightsquigarrow \theta'}{\vdash_{\mathtt{r}}} x : \{\alpha \in \mathtt{R}\}}$$

$$\text{Rf}\ \frac{\begin{array}{l} f \in \mathfrak{F} \text{ is continuous on } \mathrm{Dom}(\theta'_1 \wedge \ldots \wedge \theta'_n)^{\alpha_1 \cdots \alpha_n} \\ f(\mathrm{Dom}(\theta'_1 \wedge \ldots \wedge \theta'_n)^{\alpha_1 \cdots \alpha_n}) \subseteq \mathrm{Dom}(\theta')^{\beta} \end{array} \qquad \Gamma \overset{\theta \rightsquigarrow \theta'_i}{\vdash_{\mathtt{r}}} t_i : \{\alpha_i \in \mathtt{R}\}}{\Gamma \overset{\theta \rightsquigarrow \theta'}{\vdash_{\mathtt{r}}} \underline{f}(t_1 \ldots t_n) : \{\beta \in \mathtt{R}\}}$$

$$\text{abs}\ \frac{\Gamma, x_1 : T_1, \ldots, x_n : T_n \overset{\psi(\eta)}{\vdash_{\mathtt{r}}} t : T \qquad \models \psi_1 \wedge \psi_2 \Rightarrow \psi}{\Gamma \overset{\psi_2}{\vdash_{\mathtt{r}}} \lambda(x_1, \ldots, x_n).t : (T_1, \ldots, T_n) \overset{\psi_1(\eta)}{\to} T}$$

$$\text{app}\ \frac{\begin{array}{l} (\Gamma \overset{\phi}{\vdash_{\mathtt{r}}} s_i : H_i)_{1 \le i \le m} \\ \Gamma \overset{\phi}{\vdash_{\mathtt{r}}} t : (H_1, \ldots, H_m, F_1, \ldots, F_n) \overset{\theta(\eta)}{\to} T \end{array} \qquad \begin{array}{l} \models \theta_1 \wedge \ldots \wedge \theta_n \Rightarrow \theta \\ (\Gamma \overset{\phi \rightsquigarrow \theta_j}{\vdash_{\mathtt{r}}} p_j : F_j)_{1 \le j \le m} \end{array}}{\Gamma \overset{\phi(\eta)}{\vdash_{\mathtt{r}}} t(s_1, \ldots, s_m, p_1, \ldots, p_m) : T}$$

The formula $\psi(\eta)$ should be read as $\psi$ when $T$ is a higher-order type, and as $\psi \rightsquigarrow \eta$ when $T$ is a ground type.

Fig. 4: Typing Rules

*Example 6.* Let $f : \mathbb{R} \to \mathbb{R}$ be the function defined as: $f(x) = \begin{cases} -x \text{ if } x < 0 \\ x + 1 \text{ otherwise} \end{cases}$. Observe that we can actually regard $f$ as represented by the program in Figure 3a—but we consider it as a primitive function in $\mathfrak{F}$ for the time being, since we have not introduced the typing rule for the if-then-else construct, yet. Consider the program:

$$t = \lambda(x, y).\underline{f}(\underline{min}(x, y)).$$

We see that $[\![t]\!] : \mathbb{R}^2 \to \mathbb{R}$ is continuous on the set $\{(x, y) \mid x \ge 0 \wedge y \ge 0\}$, and that, moreover, the image of $f$ on this set is contained on $[1, +\infty)$. Using the rules in Figure 4, the fact that $f$ is continuous on $\mathbb{R}_{\ge 0}$, and that *min* is continuous on $\mathbb{R}^2$, we see that our refined type system allows us to prove $t$ to be continuous in the considered domain, i.e.:

$$\vdash_{\mathtt{r}} t : (\{\alpha \in \mathtt{R}\}, \{\beta \in \mathtt{R}\}) \overset{(\alpha \ge 0 \wedge \beta \ge 0) \rightsquigarrow (\gamma \ge 1)}{\to} \{\gamma \in \mathtt{R}\}.$$

### 6.3 Typing Conditionals

We now look at the rule for the if-then-else construct: as can be seen in the two programs in Figure 3, the use of conditionals *may* or *may not* induce discontinuity points. The crux here is the behaviour of the two branches at the

*discontinuity points of the guard function.* In the two programs represented in Figure 3, we see that the only discontinuity point of the guard is in $x = 0$. However, in Figure 3b the two branches return the same value in 0, and the resulting program is thus continuous at $x = 0$, while in Figure 3a the two branches do not coincide in 0, and the resulting program is discontinuous at $x = 0$. We can generalize this observation: for the program `if` $t$ `then` $s$ `else` $p$ to be continuous, we need the branches $s$ and $p$ to be continuous respectively on the domain where $t$ is 1, and on the domain where $t$ is 0, and moreover we need $s$ and $p$ to be continuous *and to coincide* on the points where $t$ is not continuous. Similarly to the logical system designed by Chaudhuri et al [18], the coincidence of the branches in the discontinuity points is expressed as a set of logical rules by way of *observational equivalence*. It should be observed that such an equivalence check is less problematic for first-order programs than it is for higher-order one (the authors of [18] are able to actually check observational equivalence through an SMT solver). On the other hand, various notions of equivalence which are included in contextual equivalence and sometimes coincide with it (e.g., applicative bisimilarity, denotational semantics, or logical relations themselves) have been developed for higher-order languages, and this starts to give rise to actual automatic tools for deciding contextual equivalence [38].

We give in Figure 5 the typing rule for conditionals. The conclusion of the rule guarantees the continuity of the program `if` $t$ `then` $s$ `else` $p$ on a domain specified by a formula $\theta$. The premises of the rule ask for formulas $\theta_q$ for $q \in \{t, s, p\}$ that specify continuity domains for the programs $t$, $s$, $p$, and ask also for two additional formulas $\theta_{(t,0)}$ and $\theta_{(t,1)}$ that specify domains where the value of the guard $t$ is 0 and 1, respectively. The target formula $\theta$, and the formulas $(\theta_q)_{q\in\{t,s,p,(t,1),(t,0)\}}$ are related by two side-conditions. Side-condition (1) consists of the following four distinct requirements, that must hold for every point $a$ in the truth domain of $\theta$: i) $a$ is in the truth domain of at least one of the two formulas $\theta_t$, $\theta_s$; ii) if $a$ is not in $\theta_{(t,1)}$ (i.e., we have no guarantee that $t$ will return 1 at point $a$, meaning that the program $p$ *may* be executed) then $a$ must be in the continuity domain of $p$; iii) a condition symmetric to the previous one, replacing 1 by 0, and $p$ by $s$; iv) all points of possible discontinuity (i.e. the points $a$ such that $\theta^t$ does not hold) must be in the continuity domain of both $s$ and $p$, and as a consequence both $\theta^s$ and $\theta^p$ must hold there. The side-condition (2) uses *typed contextual equivalence* $\equiv^{ctx}$ between terms to express that the two programs $s$ and $p$ must coincide on all inputs such that $\theta_t$ does not hold–i.e. that are not in the continuity domain of $t$. Observe that typed context equivalence here is defined with respect to the system of *simple types.*

**Notation 1.** *We use the following notations in Figure 5. When $\Gamma$ is a typing environement, we write $G\Gamma$ and $H\Gamma$ for the ground and higher-order parts of $\Gamma$, respectively. Moreover, suppose we have a ground refined typing environment $\Theta = x_1 : \{\alpha_1 \in \mathtt{R}\}, \ldots, x_n : \{\alpha_n \in \mathtt{R}\}$: we say that a logical assignment $\sigma$ is* compatible with $\Theta$ *when* $\{\alpha_i \mid 1 \leq i \leq n\} \subseteq \mathsf{supp}(\sigma)$. *When it is the case, we build in a natural way the* substitution associated to $\sigma$ along $\Theta$ *by taking* $\sigma^{\Theta}(x_i) = \underline{\sigma(\alpha_i)}$.

If $$\frac{\begin{array}{c}\Gamma \overset{\theta_t \rightsquigarrow (\beta=0 \vee \beta=1)}{\vdash_{\mathtt{r}}} t : \{\beta \in \mathtt{R}\} \\ \Gamma \overset{\theta_{(t,0)} \rightsquigarrow (\beta=0)}{\vdash_{\mathtt{r}}} t : \{\beta \in \mathtt{R}\} \\ \Gamma \overset{\theta_{(t,1)} \rightsquigarrow (\beta=1)}{\vdash_{\mathtt{r}}} t : \{\beta \in \mathtt{R}\}\end{array} \qquad \Gamma \overset{\theta_s(\eta)}{\vdash_{\mathtt{r}}} s : T \qquad \Gamma \overset{\theta_p(\eta)}{\vdash_{\mathtt{r}}} p : T}{\Gamma \overset{\theta(\eta)}{\vdash_{\mathtt{r}}} \mathtt{if}\ t\ \mathtt{then}\ s\ \mathtt{else}\ p : T} \quad (1), (2)$$

Again, the formula $\psi(\eta)$ should be read as $\psi$ when $T$ is a higher-order type, and as $\psi \rightsquigarrow \eta$ when $T$ is a ground type. The side-conditions (1), (2) are given as:

1. $\models \theta \Rightarrow \Big( (\theta^s \vee \theta^p) \wedge (\theta^{(t,1)} \vee \theta^p) \wedge (\theta^{(t,0)} \vee \theta^s) \wedge (\theta_t \vee (\theta_s \wedge \theta_p)) \Big)$.
2. For all logical assignment $\sigma$ compatible with $G\Gamma$, $\sigma \models \theta \wedge \neg\theta_t$ implies $H\Gamma \vdash s\sigma^{G\Gamma} \equiv^{ctx} p\sigma^{G\Gamma}$.

Fig. 5: Typing Rules for the if-then-else construct

*Example 7.* Using our if-then-else typing rule, we can indeed type the program in Figure 3b as expected:

$$\vdash \lambda x.\mathtt{if}\ x < 0\ \mathtt{then}\ 1\ \mathtt{else}\ x + 1 : \{\alpha \in \mathtt{R}\} \overset{\top \rightsquigarrow \top}{\rightarrow} \{\beta \in \mathtt{R}\}.$$

### 6.4 Open-logical Predicates for Refinement Types

Our goal in this section is to show the correctness of our refinement type systems, that we state below.

**Theorem 3.** *Let $t$ be any program such that:*

$$x_1 : \{\alpha_1 \in \mathtt{R}\}, \ldots, x_n : \{\alpha_n \in \mathtt{R}\} \overset{\theta \rightsquigarrow \theta'}{\vdash_{\mathtt{r}}} t : \{\beta \in \mathtt{R}\}.$$

*Then it holds that:*

- $[\![t]\!](Dom(\theta)^{\alpha_1,\ldots,\alpha_n}) \subseteq Dom(\theta')^{\beta}$*;*
- $[\![t]\!]$ *is sequentially continuous on* $Dom(\theta)^{\alpha_1,\ldots,\alpha_n}$ *.*

As a first step, we show that our if-then-else rule is reasonable, i.e. that it behaves well with primitive functions in $\mathfrak{F}$. More precisely, if we suppose that the functions $f, g_0, g_1$ are such that the premises of the if-then-else rule hold, then the program $\mathtt{if}\ \underline{f}(x_1, \ldots, x_n)\ \mathtt{then}\ \underline{g_1}(x_1, \ldots, x_n)\ \mathtt{else}\ \underline{g_0}(x_1, \ldots, x_n)$ is indeed continuous in the domain specified by the conclusion of the rule. This is precisely what we prove in the following lemma.

**Lemma 4.** *Let $f, g_0, g_1 : \mathbb{R}^n \to \mathbb{R}$ be functions in $\mathfrak{F}$, and $\Theta = x_1 : \{\alpha_1 \in \mathtt{R}\}, \ldots, x_n : \{\alpha_n \in \mathtt{R}\}$. We denote $\boldsymbol{\alpha}$ the list of logical variables $\alpha_1, \ldots, \alpha_n$. We consider logical formulas $\theta$ and $\theta_f, \theta_{(f,0)}, \theta_{(f,1)}, \phi_{g_0}, \phi_{g_1}$ that have their logical variables in $\boldsymbol{\alpha}$, and such that:*

1. *$f$ is continuous on $Dom(\theta)^{\boldsymbol{\alpha}}$ with $f(Dom(\theta_f)^{\boldsymbol{\alpha}}) \subseteq \{0,1\}$ and $f(Dom(\theta_{(f,b)})^{\boldsymbol{\alpha}}) \subseteq \{b\}$ for $b \in \{0,1\}$.*
2. *$g_0$ and $g_1$ are continuous on $Dom(\phi_{g_0})^{\boldsymbol{\alpha}}$, and $Dom(\phi_{g_1})^{\boldsymbol{\alpha}}$ respectively, and $(\alpha_1 \mapsto a_1, \ldots, \alpha_n \mapsto a_n) \models \theta \wedge \neg\theta_f$ implies $g_0(a_1, \ldots, a_n) = g_1(a_1, \ldots, a_n)$;*
3. *$\models \theta \Rightarrow \left((\phi_{g_1} \vee \phi_{g_0}) \wedge (\theta_{(f,0)} \vee \phi_{g_1}) \wedge (\theta_{(f,1)} \vee \phi_{g_0}) \wedge (\theta_f \vee (\phi_{g_0} \wedge \phi_{g_1}))\right)$.*

*Then it holds that:*

$$[\![\overline{\Theta} \vdash \texttt{if } \underline{f}(x_1, \ldots, x_n) \texttt{ then } \underline{g_1}(x_1, \ldots, x_n) \texttt{ else } \underline{g_0}(x_1, \ldots, x_n) : \texttt{R}]\!]$$

*is continuous on $Dom(\theta)^{\boldsymbol{\alpha}}$.*

*Proof.* The proof can be found in the extended version [7]. □

Similarly to what we did in Section 4, we are going to show Theorem 3 by way of a logical predicate. Recall that the logical predicate we defined in Section 4 consists actually of *three* kind of predicates—all defined in Definition 1 of Section 4: $\mathcal{F}_\tau^\Theta$, $\mathcal{F}_\Gamma^\Theta$, $\mathcal{F}_\tau^{\Theta,\Gamma}$, where $\Theta$ ranges over ground typing environments, $\Gamma$ ranges over arbitrary environments, and $\tau$ is a type. The first predicate $\mathcal{F}_\tau^\Theta$ contains admissible terms $t$ of type $\Theta \vdash t : \tau$, the second predicate $\mathcal{F}_\Gamma^\Theta$ contains admissible substitutions $\gamma$ that associate to every $(x : \tau)$ in $\Gamma$ a term of type $\tau$ under the typing context $\Theta$, and the third predicate $\mathcal{F}_\tau^{\Theta,\Gamma}$ contains admissible terms $t$ of type $\Gamma, \Theta \vdash t : \tau$.

Here, we need to adapt the three kinds of logical predicates to a refinement scenario: first, we replace $\tau$ and $\Theta$, $\Gamma$ with refinement types and refined typing contexts respectively. Moreover, for technical reasons, we also need to *generalize* our typing contexts, by allowing them to be annotated with any subset of $\mathbb{R}^n$ instead of restricting ourselves to those subsets generated by logical formulas. Due to this further complexity, we split our definition of logical predicates into two: we first define the counterpart of the ground typing context predicate $\mathcal{F}_\tau^\Theta$ in Definition 4, then the counterpart of the predicate for substitutions $\mathcal{F}_\Gamma^\Theta$ and the counterpart of the predicates $\mathcal{F}_\tau^{\Theta,\Gamma}$ for higher-order typing environment in Definition 5.

Let us first see how we can adapt the predicates $\mathcal{F}_\tau^\Theta$ to our refinement types setting. Recall that in Section 4, we defined the predicate $\mathcal{F}_{\texttt{R}}^\Theta$ as the collection of terms $t$ such that $\Theta \vdash t : \texttt{R}$, and its semantics $[\![\Theta \vdash t : \texttt{R}]\!]$ belongs to $\mathfrak{F}$. As we are interested in local continuity properties, we need to build a predicate expressing local continuity constraints. Moreover, in order to be consistent with our two arrow constructs and our two kinds of typing judgments, we actually need to consider also *two* kinds of logical predicates, depending on whether the target type we consider is a real type or an higher-order type. We thus introduce the following logical predicates:

$$\mathcal{C}(\Theta, X \rightsquigarrow \phi, F); \qquad \mathcal{C}(\Theta, X, H);$$

where $\Theta$ is a ground typing environment, $X$ is a subset of $\mathbb{R}^n$, $\phi$ is a logical formula, and, as usual, $F$ ranges over the real refinements types, while $H$ ranges over the higher-order refinement types. As expected, $X$ and $\phi$ are needed to encode continuity constraints inside our logical predicates.

**Definition 4.** *Let $\Theta$ be a* ground *typing context of length $n$, $F$ and $H$ refined ground type and higher-order type, respectively. We define families of predicates on terms $\mathcal{C}(\Theta, Y \rightsquigarrow \phi, F)$ and $\mathcal{C}(\Theta, Y, H)$, with $Y \subseteq \mathbb{R}^n$ and $\phi$ a logical formula, as specified in Figure 6.*

- For $F = \{\alpha \in \mathtt{R}\}$ we take:

$$\mathcal{C}(\Theta, Y \rightsquigarrow \psi, F) := \{t \mid x_1 : \mathtt{R}, \ldots, x_n : \mathtt{R} \vdash t : \mathtt{R}, \\ [\![t]\!](Y) \subseteq \mathrm{Dom}(\psi)^{\alpha} \wedge [\![t]\!] \text{ continuous over } Y\}.$$

- if $H$ is an arrow type of the form $H = (H_1, \ldots, H_m, \{\alpha_1 \in \mathtt{R}_1\}, \ldots, \{\alpha_p \in \mathtt{R}\}) \overset{\psi(\eta)}{\to} T$:

$$\begin{aligned} \mathcal{C}(\Theta, Y, H) := \{t \mid x_1 : \mathtt{R}, \ldots, x_n : \mathtt{R} \vdash t : \overline{H}, \\ \forall Z, \forall \boldsymbol{s} = (s_1, \ldots, s_m) \text{ with } s_i \in \mathcal{C}(\Theta, Z, H_i), \\ \forall \boldsymbol{p} = (p_1, \ldots p_p), \forall \psi^j \text{ with } \models \psi^1 \wedge \ldots \wedge \psi^p \Rightarrow \psi, \\ \text{and } p_j \in \mathcal{C}(\Theta, Z \rightsquigarrow \psi^j, \{\alpha_j \in \mathtt{R}\}), \\ \text{it holds that } t(\boldsymbol{s}, \boldsymbol{p}) \in \mathcal{C}(\Theta, (Y \cap Z)(\eta), T)\}, \end{aligned}$$

where as usual we should read $\psi(\eta) = \psi$, $(Y \cap Z)(\eta) = Y \cap Z$ when $T$ is higher-order, and $\psi(\eta) = \psi \rightsquigarrow \eta$, $(Y \cap Z)(\eta) = (Y \cap Z) \rightsquigarrow \eta$ when $T$ is an annnotated real type.

Fig. 6: Open Logical Predicates for Refinement Types.

*Example 8.* We illustrate Definition 4 on some examples. We denote by $B^\circ$ the open unit ball in $\mathbb{R}^2$, i.e. $B^\circ = \{(a, b) \in \mathbb{R}^2 \mid a^2 + b^2 < 1\}$. We consider the ground typing context $\Theta = x_1 : \{\alpha_1 \in \mathtt{R}\}, x_2 : \{\alpha_2 \in \mathtt{R}\}$.

- We look first at the predicate $\mathcal{C}(\Theta, B^\circ \rightsquigarrow (\beta > 0), \{\beta \in \mathtt{R}\})$. It consists of all programs $x_1 : \mathtt{R}, x_2 : \mathtt{R} \vdash t : \mathtt{R}$ such that $[\![x_1 : \mathtt{R}, x_2 : \mathtt{R} \vdash t : \mathtt{R}]\!]$ is continuous on the open unit ball, and takes only strictly positive values there.
- We look now at an example when the target type $T$ is *higher-order*. We take $H = \{\beta_1 \in \mathtt{R}\} \overset{(\beta_1 \geq 0) \rightsquigarrow (\beta_2 \geq 0)}{\to} \{\beta_2 \in \mathtt{R}\}$, and we look at the logical predicate $\mathcal{C}(\Theta, B^\circ, H)$. We are going to show that the latter contains, for instance, the program:

$$t = \lambda w.\underline{f}(w, x_1^2 + y_1^2) \qquad \text{where } f(w, a) = \frac{w}{1 - a} \text{ if } a < 1; 0 \text{ otherwise.}$$

Looking at Figure 6, we see that it is enough to check that for any $Y \subseteq \mathbb{R}^2$ and any $s \in \mathcal{C}(\Theta, Y \rightsquigarrow (\beta_1 \geq 0), \{\beta_1 \in \mathtt{R}\})$, it holds that:

$$ts \in \mathcal{C}(\Theta, B^\circ \cap Y \rightsquigarrow (\beta_2 \geq 0), \{\beta_2 \in \mathtt{R}\}).$$

Our overall goal—in order to prove Theorem 3—is to show the counterpart of the Fundamental Lemma from Section 4 (i.e. Lemma 1), which states that the logical predicate $\mathcal{F}_{\mathtt{R}}^{\Theta}$ contains all well-typed terms. This lemma only talks about the logical predicates for *ground typing contexts*, so we can state it as of now, but its proof is based on the fact that we dispose of the *three* predicates. Observe that from there, Theorem 3 follows just from the definition of the logical predicates on base types. Similarly to what we did for Lemma 1 in Section 4, *proving* it requires to define the logical predicates for *substitutions* and *higher-order typing contexts*. We do this in Definition 5 below. As before, they consist in an adaptation to our refinement types framework of the open logical predicates $\mathcal{F}_{\Theta}^{\Gamma}$ and $\mathcal{F}_{\tau}^{\Theta,\Gamma}$ of Section 4: as usual, we need to add continuity annotations, and distinguish whether the target type is a ground type or an higher-order type.

**Notation 2.** *We need to first introduce the following notation: let $\Gamma$, $\Theta$ be two ground non-refined typing environments of length $m$ and $n$ respectively–and with disjoint support. Let $\gamma : \mathsf{supp}(\Gamma) \to \{t \mid \overline{\Theta} \vdash t : \mathtt{R}\}$ be a substitution. We write $[\![\gamma]\!]$ for the real-valued function:*

$$\begin{aligned} [\![\gamma]\!] :& \mathbb{R}^n \to \mathbb{R}^{n+m} \\ & \boldsymbol{a} \mapsto (\boldsymbol{a}, [\![\gamma(x_1)]\!](\boldsymbol{a}), \ldots, [\![\gamma(x_m)]\!](\boldsymbol{a})) \end{aligned}$$

**Definition 5.** *Let $\Theta$ be a ground typing environment of length $n$, and $\Gamma$ an arbitrary typing environment. We note $n$ and $m$ the lengths of respectively $\Theta$ and $G\Gamma$.*

- *Let $Z \subseteq \mathbb{R}^n, W \subseteq \mathbb{R}^{n+m}$. We define $\mathcal{C}(\Theta, Z \rightsquigarrow W, \Gamma)$ as the set of those substitutions $\gamma : \mathsf{supp}(\Gamma) \to \{t \mid \overline{\Theta} \vdash t : \mathtt{R}\}$ such that:*
  - *$\forall (x : H) \in H\Gamma$, $\gamma(x) \in \mathcal{C}(\Theta, Z, H)$,*
  - *$[\![\gamma_{|\, G\Gamma}]\!] : \mathbb{R}^n \to \mathbb{R}^{n+m}$ sends continuously $Z$ into $W$;*
- *Let $W \subseteq \mathbb{R}^{n+m}$, $F = \{\alpha \in \mathtt{R}\}$ an annotated real type, and $\psi$ a logical formula with $Vars(\psi) \subseteq \{\alpha\}$. We define:*

$$\begin{aligned} \mathcal{C}((\Gamma;\Theta), W \rightsquigarrow \psi, F) := \{t \mid & \overline{\Gamma,\Theta} \vdash t : \mathtt{R} \\ & \wedge \forall X \subseteq \mathbb{R}^n, \forall \gamma \in \mathcal{C}(\Theta, X \rightsquigarrow W, \Gamma),\ t\gamma \in \mathcal{C}(\Theta, X \rightsquigarrow \psi, F)\}. \end{aligned}$$

- *Let $W \subseteq \mathbb{R}^{n+m}$, and $H$ an higher-order refined type. We define :*

$$\begin{aligned} \mathcal{C}((\Gamma;\Theta), W, H) := \{t \mid & \overline{\Gamma,\Theta} \vdash t : \overline{H} \\ & \wedge \forall X \subseteq \mathbb{R}^n, \forall \gamma \in \mathcal{C}(\Theta, X \rightsquigarrow W, \Gamma).\ t\gamma \in \mathcal{C}(\Theta, X, H)\}. \end{aligned}$$

*Example 9.* We illustrate Definition 5 on an example. We consider the same context $\Theta$ as in Example 8, i.e. $\Theta = x_1 : \{\alpha_1 \in \mathtt{R}\}, x_2 : \{\alpha_2 \in \mathtt{R}\}$, and we take $\Gamma = x_3 : \{\alpha_3 \in \mathtt{R}\}, z : H$, with $H = \{\beta_1 \in \mathtt{R}\} \overset{(\beta_1 \geq 0) \rightsquigarrow (\beta_2 \geq 0)}{\to} \{\beta_2 \in \mathtt{R}\}$. We are interested in the following logical predicate for substitution:

$$\mathcal{C}(\Theta, B^\circ \rightsquigarrow \{(v, |v|) \mid v \in B^\circ)\}, \Gamma)$$

where the norm of the couple $(a, b)$ is taken as: $|(a, b)| = \sqrt{a^2 + b^2}$. We are going to build a substitution $\gamma : \{x_3, z\} \to \Lambda_{\mathfrak{F}}^{\times,\to,\mathtt{R}}$ that belongs to this set. We take:

- $\gamma(z) = \lambda w.\underline{f}(w, x_1^2 + x_2^2)$ where $f(w, a) = \frac{w}{1-a}$ if $a < 1; 0$ otherwise.
- $\gamma(x_3) = \underline{(\sqrt{\cdot})}(x_1^2 + x_2^2)$.

We can check that the requirements of Definition 5 indeed hold for $\gamma$:

- $\gamma(z) \in \mathcal{C}(\Theta, B^\circ, H)$—see Example 8;
- $[\![\gamma_{|G\Gamma}]\!] : \mathbb{R} \times \mathbb{R} \to \mathbb{R}^3$ is continuous on $B^\circ$, and moreover sends $B^\circ$ into $\{(v, |v|) \mid v \in B^\circ)\}$. Looking at our definition of the semantics of a substitution, we see that $[\![\gamma_{|G\Gamma}]\!](a, b) = (a, b, |(a, b)|)$, thus the requirements above hold.

**Lemma 5 (Fundamental Lemma).** *Let $\Theta$ be a ground typing context, and $\Gamma$ an arbitrary typing context–thus $\Gamma$ can contain both ground type variables and non-ground type variables.*

- *Suppose that $\Gamma, \Theta \overset{\theta \rightsquigarrow \eta}{\vdash_{\mathtt{r}}} t : F$: then $t \in \mathcal{C}(\Gamma; \Theta, Dom(\theta) \rightsquigarrow \eta, F)$.*
- *Suppose that $\Gamma, \Theta \overset{\theta}{\vdash_{\mathtt{r}}} t : H$: then $t \in \mathcal{C}(\Gamma; \Theta, Dom(\theta), H)$.*

*Proof Sketch.* The proof is by induction on the derivation of the refined typing judgment. Along the lines, we need to show that our logical predicates play well with the underlying denotational semantics, but also with logic. The details can be found in the extended version [7]. □

From there, we can finally prove the main result of this section, i.e. Theorem 3, that states the correctness of our refinement type system. Indeed, Lemma 5 has Theorem 3 as a corollary: from there it is enough to look at the definition of the logical predicate for first-order programs to finally show the correctness of our type system.

## 7 Related Work

Logical relations are certainly one of the most well-studied concepts in higher-order programming language theory. In their unary version, they have been introduced by Tait [54], and further exploited by Girard [33] and Tait [55] himself in giving strong normalization proofs for second-order type systems. The relational counterpart of realizability, namely logical relations proper, have been introduced by Plotkin [48], and further developed along many different axes, and in particular towards calculi with fixpoint constructs or recursive types [3,4,2], probabilistic choice [14], or monadic and algebraic effects [34,11,34]. Without any hope to be comprehensive, we may refer to Mitchell's textbook on programming language theory for a comprehensive account about the earlier, classic definitions [43], or to aforementioned papers for more recent developments.

Extensions of logical relations to open terms have been introduced by several authors [39,47,30,53,15] and were explicitly referred to as *open logical relations* in [59]. However, to the best of the authors' knowledge, all the aforementioned works use open logical relations for specific purposes, and do not investigate their applicability as a general methodology.

Special cases of our Containment Theorem can be found in many papers, typically as auxiliary results. As already mentioned, an example is the one of higher-order polynomials, whose first-order terms are proved to compute proper polynomials in many ways [40,5], none of them in the style of logical relations. The Containment Theorem itself can be derived by a previous result by Lafont [41] (see also Theorem 4.10.7 in [24]). Contrary to such a result, however, our proof of the Containment Theorem is entirely syntactical and consists of a straightforward application of open logical relations.

Algorithms for automatic differentiation have recently been extended to higher-order programming languages [50,46,51,42,45], and have been investigated from a semantical perspective in [16,1] relying on insights from linear logic and denotational semantics. In particular, the work of Huot et al. [37] provides a denotational proof of correctness of the program transformation of [50] that we have studied in Section 5.

Continuity and robustness analysis of imperative first-order programs by way of program logics is the topic of study of a series of papers by Chaudhuri and co-authors [19,18,20]. None of them, however, deal with higher-order programs.

## 8 Conclusion and Future Work

We have showed how a mild variation on the concept of a logical relation can be fruitfully used for proving both predicative and relational properties of higher-order programming languages, when such properties have a first-order, rather than a ground "flavor". As such, the added value of this contribution is not much in the technique itself, but in showing how it is extremely useful in heterogeneous contexts, this way witnessing the versatility of logical relations.

The three case studies, and in particular the correctness of automatic differentiation and refinement type-based continuity analysis, are given as proof-of-concepts, but this does not mean they do not deserve to be studied more in depth. An example of an interesting direction for future work is the extension of our correctness proof from Section 5 to backward propagation differentiation algorithms. Another one consists in adapting the refinement type system of Section 6.1 to deal with differentiability. That would of course require a substantial change in the typing rule for conditionals, which should take care of checking not only continuity, but also differentiability at the critical points. It would also be interesting to implement the refinement type system using standard SMT-based approaches. Finally, the authors plan to investigate extensions of open logical relations to non-normalizing calculi, as well as to non-simply typed calculi (such as calculi with polymorphic or recursive types).
